# Health-Related Coping Behaviors Among Parents of Children with Inborn Errors of Metabolism: A Survey by Dietary Therapy, Child Age, and Diagnostic Category

**DOI:** 10.3390/ijns12020032

**Published:** 2026-05-06

**Authors:** Yuko Matsumoto, Yuko Kushihashi, Akiko Suwa, Go Tajima

**Affiliations:** 1 Department of Nursing, Kagawa Prefectural University of Health Sciences, Takamatsushi 761-0123, Kagawa, Japan; suwa.aki.xa@yokohama-cu.ac.jp; 2 Department of Nursing, Wakayama Postgraduate School of Nursing, Tokyo Healthcare University, Wakayamashi 640-8269, Wakayama, Japan; y-kushihashi@thcu.ac.jp; 3 Division of Neonatal Screening, Research Institute, National Center for Child Health and Development, Setagaya-ku, Tokyo 157-8535, Japan; tajima-g@ncchd.go.jp

**Keywords:** newborn mass screening, inborn errors of metabolism, dietary therapy, family-centered care

## Abstract

Newborn mass screening improves outcomes for inborn errors of metabolism (IEM); nonetheless, home-based dietary therapy imposes a substantial parental burden. In this study, we explored differences in parents’ health coping behaviors, assessed using the Coping Health Inventory for Parents (CHIP), based on the presence of dietary therapy, child age group, and diagnostic category. A 21-item, CHIP-based questionnaire was distributed via the JaSMIn registry to parents of children with IEM up to school age. Overall, 201 valid responses (56.1% response rate) were analyzed regarding the implementation and perceived usefulness of coping behaviors, stratified by child age, enrollment, diagnosis, and dietary therapy. Parents in the dietary-therapy group reported more coping behaviors than did those in the non-dietary-therapy group. Notably, parents of children aged 1–3 years (not yet in preschool) and those of children with organic acid metabolism disorders rated “daily home practice of treatments” as a highly useful coping behavior. Health-related coping behaviors among parents of children with IEM vary substantially according to child age and disease characteristics. Therefore, family support strategies should be tailored to specific developmental stages and treatment requirements.

## 1. Introduction

Newborn mass screening (NBS) programs aim to detect abnormalities in newborns at an early stage, enabling timely treatment that prevents the onset or progression of intellectual and physical disabilities. Advances in NBS testing methods, expansion of target disorders, and improved treatment modalities have enhanced clinical outcomes and reduced healthcare costs associated with disease prevention [1,2,3]. Consequently, inborn errors of metabolism (IEM) should be managed as chronic conditions, with long-term treatment primarily administered in the home setting.

The effectiveness of dietary therapy for IEM is well established. However, compared with medical treatment, it imposes a heavier burden on families [4]. The resultant psychosocial strain on parents providing daily dietary management is widely documented [5]. Gastrostomy may improve physical and social quality of life (QOL) [6]. However, dietary restrictions can negatively impact children’s QOL and increase parental anxiety by influencing children’s trait anxiety, prosocial behavior, and total difficulties [7]. Consequently, parents often struggle to maintain their own health while adhering to complex home-based treatment regimens for their children.

The transition to daycare represents a pivotal moment in disease management for children with chronic conditions [8]. Parental involvement in care correlates with children’s metabolic control [9] and prosocial behaviors that support social adaptation [10]. Early childhood is a period of rapid cognitive and socio-emotional development. Treatment adherence during this period becomes increasingly complex as disease management requires coordination across home and social environments, particularly upon entry into group-based care.

A systematic review indicated that elevated parenting stress among caregivers of children with chronic illness is not consistently associated with disease severity or duration, but rather with the extent of parental responsibility for treatment and general parenting demands [11]. Such stress can interfere with caregivers’ psychological adjustment and children’s adaptation, potentially compromising disease management and health outcomes.

These developmental and contextual demands may strain parents’ physical and mental health, affecting the stability of long-term home-based care. Therefore, beyond assessing subjective stress or QOL of parents, research must focus on parental coping behaviors—specifically for maintaining physical and mental health—as modifiable factors that may support long-term home-based treatment.

Qualitative research highlights that parents of children with inborn metabolic disorders employ diverse coping processes to maintain psychological stability. For instance, mothers raising children with amino acid metabolism disorders have described two contrasting coping processes for maintaining psychological stability: “passing difficulties in the duality of hope/hopelessness” and “isolation–socialization” [12]. These findings underscore that parents of children with IEM engage in various health-related coping behaviors to preserve family homeostasis.

The Coping Health Inventory for Parents (CHIP), developed by McCubbin et al., helps conceptualize such diverse coping behaviors in a systematic and clinically applicable manner. It offers a robust framework for identifying these behaviors as modifiable strategies for managing caregiving demands [13]. It comprises three subscales: (I) Family Integration, Cooperation, and an Optimistic Definition of the Situation; (II) Maintaining Social Support, Self-Esteem, and Psychological Stability; (III) Understanding the Health Care Situation Through Communication with Other Parents and Consultation with the Health Care Team. Importantly, the demands placed on parental health-related coping behaviors may vary by disease characteristics and treatment requirements. For example, compared with medication-based treatment, dietary therapy-centered management often requires preparation of three daily meals in addition to snacks, imposing substantial time constraints. Such demands may restrict employment opportunities, social engagement, and self-care activities, thereby disproportionately influencing coping behaviors related to support, esteem, and stability (CHIP Factor II). Furthermore, disease groups with a high risk of acute metabolic decompensation (e.g., acute-type amino acid and organic acid metabolism disorders and acute-type fatty acid oxidation disorders) require constant vigilance and rapid decision-making in daily life [14]. These clinical characteristics may increase demands on coping behaviors related to integration, cooperation, and optimism (CHIP Factor I), as well as medical communication and consultation (Factor III).

Conversely, non-acute amino acid metabolism disorders and neonatal intrahepatic cholestasis caused by citrin deficiency (NICCD) are typically characterized by fewer acute episodes; their management is focused on long-term nutrition and adaptation to uncertainty [15]. Indeed, studies using the CHIP have shown that parents of children with metabolic disorders (e.g., phenylketonuria [PKU]) tend to engage less frequently in coping behaviors related to support, esteem, and stability (Factor II) than those directed toward family integration or medical communication [16,17]. Collectively, these findings highlight the importance of understanding parental health-related coping behaviors to support child condition management and overall family psychosocial stability.

In Japan, parents raising children with IEM also experience diminished QOL associated with high stress levels and low family empowerment [18]; the caregiving burden is further amplified when developmental disabilities are present [19]. Clinicians and other supporters need to understand how parents confront their physical and mental health challenges, maintain their health, and identify useful coping behaviors. Yet, no studies in Japan have specifically focused on parental health-related coping behaviors. Furthermore, as children with IEM transition from home-based, family-centered care in early childhood to group-based care in preschool and school settings, treatment feasibility and parental coping behaviors may shift; however, these changes remain insufficiently understood. In particular, Japan has a school lunch system where nurseries and schools provide meals with set menus. Consequently, people are forced to change and adjust their eating habits as they move from one social group to another.

In this study, we aimed to explore differences in parental health-related coping behaviors based on the presence of dietary therapy, child age group, and diagnostic category. The findings will provide direction for future research on family-centered care tailored to child age and disease characteristics.

In this study, “health-related coping behaviors” were operationally defined by applying the established factor structure of the CHIP, developed by McCubbin et al. [13]. Specifically, we analyzed the CHIP items reflecting parental behavior regarding maintaining physical and mental health in the context of caregiving. It encompassed the following domains: (1) Family Cohesion and Positive Beliefs, (2) Maintaining Well-being and Social Relationships, and (3) Applied Medical Understanding and Communication. This approach clarifies the analytic focus on parental health-maintenance behaviors without altering the original conceptual framework of the CHIP.

Given the exploratory nature of this study, no specific hypotheses were formulated. By examining how disease characteristics and developmental stages may relate to distinct domains of parental health-related coping behaviors, this study seeks to generate hypotheses and provide clinically meaningful insights to inform age- and condition-sensitive family-centered care strategies.

## 2. Materials and Methods

### 2.1. Study Design

As each IEM is a rare condition, accumulating evidence on patterns of parental health-related coping behaviors for specific disorders and developmental stages is challenging. Therefore, this study was designed as an exploratory investigation to examine patterns of association between parental health-related coping behaviors and clinically and developmentally relevant characteristics, aiming to contribute to hypothesis generation for future research.

This survey was prospectively planned as a component of a broader research project examining the lived experiences and care-related circumstances of families of children with IEM. Although the overarching survey covered multiple child-centered care-related domains, the CHIP was included to assess parental health-related coping behaviors, which are distinct from the child-centered domains. Although these components addressed different research questions, they were administered concurrently for practical reasons, including reducing respondent burden and minimizing time and costs. The CHIP-related analyses reported herein were specified a priori as an independent research objective and do not represent a post hoc or secondary analysis of data collected for another purpose. Given the conceptual independence of parental coping behaviors from child-focused care variables, the results are reported separately from other components of the broader project.

### 2.2. Data Collection

#### 2.2.1. Study Participants

Following approval for collaboration from the Japan Registration System for Metabolic and Inherited Diseases (JaSMIn), the survey materials were distributed by mail by the JaSMIn secretariat. They were sent to parents of children up to school age, who were diagnosed with disorders included in the NBS program in Japan. At the time of data collection, the program encompassed approximately 20 inherited metabolic disorders, including primary and secondary target conditions detectable by tandem mass spectrometry (e.g., amino acid metabolism disorders, urea cycle disorders, organic acid metabolism disorders, fatty acid oxidation disorders, and galactosemia) [20].

Consequently, this study targeted parents of children with suspected inherited metabolic disorders within this disease spectrum, who were undergoing or had undergone diagnostic evaluation, irrespective of whether the identification was through newborn screening or based on clinical presentation. As the diagnostic process for inherited metabolic disorders was often ongoing, eligibility was not limited to children with a confirmed diagnosis at the time of survey. Conversely, disorders primarily classified as endocrine conditions (e.g., congenital hypothyroidism or congenital adrenal hyperplasia) were excluded, as they fall outside the conceptual scope of metabolic disorders addressed herein.

Participation was limited to parents of children up to school age, as this study focused on parental caregiving and health-related coping rather than transition-related issues associated with adolescent self-management. The questionnaire was completed by the primary caregiver responsible for the child’s daily disease management. This approach was adopted to ensure that reported coping behaviors reflected comparable levels of caregiving involvement across participants.

#### 2.2.2. Data Collection Procedures

Data were collected between 20 December 2022 and 20 January 2023, utilizing an anonymous, self-administered questionnaire delivered by mail and via a web-based platform. Participants were permitted to select their preferred response mode.

Survey materials, including a paper questionnaire and a quick response (QR) code linked to the web-based questionnaire, were mailed to eligible participants by the JaSMIn secretariat. Identifying data required for distribution (e.g., names and mailing addresses) was managed exclusively by JaSMIn and was not shared with the research team. Returned questionnaires contained no personal identifiers; the research team managed only survey data, including disease category, child age, respondent relationship, and item responses. As the survey was anonymous, information on nonrespondents was not available. Accordingly, no reminders were sent, and no follow-up contacts or additional recruitment attempts were made.

Each QR code corresponded to a unique, single-use URL, preventing duplicate responses across paper and web-based modes. Upon submission of a completed paper questionnaire, it was confirmed that the corresponding web-based URL remained unused, thereby preventing duplicate responses while maintaining anonymity.

Returned questionnaires were screened for internal consistency of basic demographic information (e.g., discrepancies between reported age and educational setting), and responses with manifest inconsistencies were excluded. For the CHIP, questionnaires with missing responses surpassing 10% of the total items were excluded from the analysis. When responses were missing for fewer than 10% of CHIP items, those items were retained as missing data without imputation. As the missing responses were not concentrated in specific items, analyses were conducted on the responses for all CHIP items.

### 2.3. Survey Measures

#### 2.3.1. Basic Attributes

Information was collected regarding parents’ relationship with the child, child’s age and preschool/school enrollment status, number of siblings, birth order, diagnosis, and whether dietary therapy was required. Given the nature of this study, the number of questionnaire items was kept to a minimum to reduce the burden on respondents and improve the response rate. Therefore, items commonly used to assess socioeconomic factors, such as educational attainment, household income, and social support, were not included.

In Japan, the average literacy level is among the highest across participating countries, and disparities in literacy associated with educational attainment are relatively small, compared with those in other countries [21]. Furthermore, in addition to universal health insurance, which ensures access to healthcare, medical expense subsidies for children are implemented nationwide at the municipal level [22]. Therefore, the economic impact on daily life associated with the use of pediatric healthcare is considered to be mitigated to some extent. Based on these considerations, the priority of these socioeconomic indicators was judged to be relatively low in this study.

#### 2.3.2. Measures of Parental Health-Related Coping Behaviors

The CHIP delineates characteristics of parental health-related coping behaviors as parents adapt to adverse circumstances [23]. Data were collected regarding the implementation status of each coping behavior (“Choose not to,” “Not possible,” or “Used”). For respondents who selected “Used,” the perceived usefulness of each behavior was evaluated using a four-point Likert scale. This scale has been employed among parents of children with chronic illnesses [16,24,25], and its internal consistency and validity have been established [13,25,26]. However, for families experiencing substantial burdens related to medical care or dietary therapy, responding to all 45 items may be onerous.

In this study, informed by prior research and cognitive debriefing conducted in collaboration with representatives of a family support organization, we ultimately selected 21 items aligned with the study objectives (Table 1). At the time of the study, the Japanese version of the CHIP was not available; therefore, the items were translated by the research team through iterative discussion until consensus was reached. The translated items were then reviewed by two individuals with lived experience to ensure clarity and consistency of interpretation. During cognitive debriefing, the items were reviewed through a stepwise process. First, items that were considered overly abstract or difficult to answer were excluded. Second, items with substantial conceptual overlap and limited distinguishability were identified. Among these, items that were judged to have greater conceptual alignment with the original subscales were retained. Finally, minor annotations were added to improve clarity and interpretability.

Cognitive debriefing strategies involving affected children and families are useful to determine the items that should be retained in instruments measuring patient-reported outcomes [27,28].

The reliability of each domain was evaluated using Cronbach’s alpha coefficient, an index of internal consistency. A value ≥ 0.70 is generally indicative of acceptable internal consistency [29]. Cronbach’s alpha coefficients were 0.85 for Domain I (9 items), 0.84 for Domain II (6 items), and 0.81 for Domain III (6 items) in the total sample. Comparable alpha values were observed in the dietary-therapy and non-dietary-therapy groups, indicating stable internal consistency across subgroups. Although the scale included mixed response formats reflecting implementation status and perceived usefulness, internal consistency was evaluated using the 4-point usefulness ratings for items endorsed as “Used”.

An exploratory or confirmatory factor analysis was not conducted; rather, items were selected while preserving the original domain structure of the CHIP, and internal consistency was examined according to the original three-domain classification to confirm that the reduced item set did not compromise structural coherence. As the primary aim of this study was not to develop a new instrument but to describe patterns of coping strategies among parents managing dietary therapy, the impact of these modifications on the scale’s structural validity was considered minimal.

### 2.4. Definition of Stratification Variables

#### 2.4.1. Definition of Dietary Therapy

Dietary therapy was analyzed as a binary variable based on physician instruction rather than on treatment intensity or adherence. The presence of dietary therapy was established if the physician’s instructions included dietary restriction, supplemental feeding, and/or the use of a specialized medical formula.

This approach was selected not only because of the limited sample size inherent to rare diseases but also because adherence to dietary management cannot be assessed consistently in clinical practice. Parental perceptions of adherence may not accurately reflect actual implementation, and the reverse may also occur. Conversely, diagnostic category and presence or absence of physician-directed dietary therapy can be assessed consistently and objectively across participants; therefore, these were utilized as stratification variables.

#### 2.4.2. Definition of Age Groups and Enrollment Status

Participants were stratified according to child age and preschool/school enrollment status, as follows: (1) ages of 1–3 years, not yet enrolled; (2) ages of 1–3 years, enrolled in preschool; (3) ages of 4–6 years, enrolled in preschool; and (4) ages of 6–12 years, enrolled in school. Infants aged 0 years were excluded from subgroup analyses because of the small sample size.

#### 2.4.3. Definition of Diagnostic Categories

For analyses by diagnostic category, participants were categorized into five diagnostic groups: (1) non-acute type amino acid metabolism disorders (AA); (2) non-acute urea cycle disorders (NICCD); (3) acute-type AA disorders (maple syrup urine disease) and acute-type urea cycle disorders (ornithine transcarbamylase deficiency, citrullinemia type I, carbamyl phosphate synthetase I deficiency, argininosuccinic aciduria); (4) organic acid metabolism disorders (OA); and (5) fatty acid metabolism disorders (FA). Non-acute AA and non-acute urea cycle disorders (NICCD) were analyzed as separate categories because the dietary therapy required for these conditions differs substantially. Non-acute AA typically involves selective protein restriction, whereas NICCD primarily requires lactose restriction. These differences were considered clinically meaningful regarding parental health-related coping behaviors. Categories with insufficient sample sizes, including carbohydrate metabolism disorders (e.g., galactosemia) and other rare or undiagnosed conditions, were excluded from subgroup analyses.

### 2.5. Statistical Analysis

Statistical comparisons focused on subgroup-based trends in parental health-related coping behaviors rather than on establishing causal relationships. Other contextual and caregiving-related factors may also influence parental coping behaviors; however, these were not examined in the present exploratory analyses.

Differences in implementation status (“Choose not to”, “Not possible”, “Used”) were examined using the correlation statistic from the Cochran–Mantel–Haenszel test as the primary indicator. For perceived usefulness, differences between parents of children with and without dietary therapy within each subgroup were assessed using the Wilcoxon rank-sum test. Differences between subgroups were examined using the Steel–Dwass test. All analyses were conducted using the Japanese version of SAS software, version 9.4 [30,31,32]. No formal adjustment for multiple comparisons was applied as the analyses were intended to identify potential patterns rather than to provide confirmatory evidence; *p*-values were therefore treated as descriptive indicators of possible associations.

This study did not include an a priori sample size calculation. Instead, the survey was designed to include all eligible families registered with the JaSMIn at the time of the study, reflecting the rarity of individual IEMs. Consequently, some subgroups comprised relatively small numbers of participants, and the absence of significant differences should not be interpreted as evidence of no association.

### 2.6. Ethical Considerations

This study was approved by the Epidemiological Research Ethics Review Committee of Hiroshima University prior to the implementation of the survey (Approval No. E2022-0145; date of approval: 11 October 2022). Participants were provided with a written explanation describing the purpose of the study, use of the collected data, assurance of anonymity, and potential burden associated with the time required to complete the questionnaire. Written informed consent was obtained from the parents before participation. Submission of the completed questionnaire and checking the consent box were regarded as provision of informed consent to participate in the study. Prior to conducting the survey, we requested and obtained cooperation from JaSMIn. All procedures were conducted in accordance with relevant ethical guidelines and regulations.

## 3. Results

### 3.1. Participant Characteristics and Response Flow

Survey materials were distributed to 369 individuals, and responses were received from 207 (56.1%). This included 107 web-based responses and 100 mailed responses. After excluding one duplicate response and five responses containing substantial inconsistencies or missing data, responses from 201 individuals (97.1%; 107 web-based and 94 mailed responses) were included in the final analysis. Among the respondents, 176 (87.6%) were mothers. The mean age of children with IEM was 6.5 years (Table 2). The most frequent diagnosis was PKU, classified as a non-acute type AA, followed by NICCD and propionic acidemia (PA), with the latter classified as an acute type of OA (Table 2). Residual analysis indicated no significant association between age group (preschool/school status) and diagnostic category. Additionally, exact tests revealed no significant associations of respondent relationship (parental status) with age group or diagnostic category, suggesting no differences in respondent relationship across the categories.

### 3.2. Results of Parental Health-Related Coping Behaviors

#### 3.2.1. Implementation and Perceived Usefulness of Health-Related Coping Behaviors by Dietary Therapy Status and Child Age/Preschool and School Enrollment Status

On the basis of the distribution of preschool and school enrollment status (Table 2), participants were stratified into age- and enrollment-based subgroups. Among children aged 1–3 years, the numbers of children receiving home care (*n* = 22) and those enrolled in childcare or kindergarten (*n* = 22) were comparable; therefore, this age group was analyzed as two separate subgroups (not-yet-enrolled and enrolled). Children aged 0 years (*n* = 6), all of whom were receiving home care, were excluded from subgroup analyses because of the small sample size. Among children aged 4–6 and 6–12 years, a negligible number of children were receiving home care only (*n* = 2 and *n* = 3, respectively). Therefore, subgroup analyses by enrollment status were not conducted in these age groups; their data were analyzed as enrolled or school-attending groups only. The magnitude of group differences varied substantially across individual coping behaviors, with effect sizes ranging from small to moderate, reflecting the heterogeneity of behaviors and limited sample sizes within subgroups.

#### 3.2.2. Implementation Status by Dietary Therapy Status and Child Age/Enrollment Status

Within the 1–3 years, not-yet-enrolled subgroup (dietary therapy *n* = 15, non-dietary therapy *n* = 7), parents in the dietary-therapy group reported a higher proportion of “Used” responses across several items, based on comparisons of implementation status between groups using the Cochran–Mantel–Haenszel test (Table S1). In particular, for “Item 43. Daily home practice of treatments,” more than 90% (14/15) of parents in the dietary-therapy group responded “Used,” whereas a markedly higher proportion of parents in the non-dietary-therapy group selected “Choose not to” (71.4%; 5/7) (Table 3).

#### 3.2.3. Perceived Usefulness by Dietary Therapy Status and Child Age/Enrollment Status

Furthermore, among parents who reported having utilized this behavior, the median usefulness score was higher in the dietary-therapy group (median = 4) than in the non-dietary-therapy group (median = 3) (Table S2).

Within the 4–6 years, preschool-enrolled subgroup, no significant differences were detected between the dietary therapy and non-dietary therapy groups regarding the implementation status of five items: “Item 2. Investing myself in my children”, “Item 5. Telling myself to be thankful”, “Item 30. Keeping in shape and groomed”, “Item 36. Building close relationships with people”, and “Item 37. Developing myself as a person” (Table 4). However, among parents who reported having employed these behaviors in the 4–6 years, preschool-enrolled subgroup (dietary therapy, *n* = 27; non-dietary therapy, *n* = 29), the median usefulness scores were lower in the dietary-therapy group (median = 2–3) than in the non-dietary-therapy group (median = 3–4), with significant differences observed across these items (Table 4).

In summary, parents in the dietary-therapy group generally reported higher overall participation in health coping behaviors, compared with the non-dietary therapy group. Among parents of children aged 1–3 years who were not yet enrolled in preschool, prescribed treatment-related behaviors were perceived as highly useful. Conversely, among parents of children aged 4–6 years enrolled in preschool, the perceived usefulness of coping behaviors pertaining to family life and self-regulation appeared modestly lower for several items.

#### 3.2.4. Implementation and Perceived Usefulness of Health-Related Coping Behaviors by Dietary Therapy Status Within Diagnostic Categories

In the analyses stratified by diagnostic category, implementation status, and perceived usefulness of health coping behaviors were compared across five diagnostic groups. As part of these analyses, between-disease comparisons restricted to parents receiving dietary therapy were conducted using the Steel–Dwass test. These comparisons suggested that the OA and NICCD groups showed different tendencies in the perceived usefulness of health-related coping behaviors. Accordingly, this section focuses in greater detail on these two diagnostic groups within the dietary-therapy group.

#### 3.2.5. Implementation Status by Dietary Therapy Status Within Diagnostic Categories

Parents in the dietary-therapy group tended to engage in a broader range of health coping behaviors, compared with those in the non-dietary therapy group, across several items. Based on comparisons of implementation status, higher proportions of “Used” responses were noted among parents in the dietary-therapy group for several coping behaviors pertaining to Factor III (Applied Medical Understanding and Communication), which comprises six items, including, for example, “Item 43. Daily home practice of treatments” and “Item 38. Talking with and learning from similar parents” (Table 5 and Table S3). This overall pattern of greater engagement in treatment- and medical-understanding-related coping behaviors among parents in the dietary-therapy group was observed across diagnostic categories. However, the specific items showing significant differences varied by disease group.

Conversely, for the two items belonging to Factor I (Family Cohesion and Positive Beliefs), significant differences by dietary therapy status were observed only in the OA group (dietary therapy, *n* = 30; non-dietary therapy, *n* = 28). Specifically, for “Item 18. Believing that the hospital has my family’s best interest in mind,” a higher proportion of parents in the dietary-therapy group responded “Used” (93.3%, 28/30), compared with the non-dietary therapy group. Conversely, for “Item 9. Believing in God,” the proportion of “Used” responses was lower in the dietary-therapy group (3.3%, 1/30) than in the non-dietary therapy group (Table 5).

#### 3.2.6. Perceived Usefulness by Dietary Therapy Status Within Diagnostic Categories

Regarding the perceived usefulness, significant differences by dietary therapy status were observed in the NICCD (dietary therapy, *n* = 20; non-dietary therapy, *n* = 4) and OA groups. Within the NICCD group, parents in the dietary-therapy group rated “Item 27. Concentrating on hobbies” lower (median = 3), compared with those in the non-dietary-therapy group (median = 4). Within the OA group, parents in the dietary-therapy group rated “Item 36. Building close relationships with people” (median = 3 vs. 4), “Item 42. Explaining family situation to our community” (median = 3 vs. 3.5), and “Item 41. Reading more about my child’s medical problem” (median = 3 vs. 3) lower than did parents in the non-dietary-therapy group (Table 6).

These comparisons were conducted among parents in the dietary-therapy group only, rather than performing within-disease comparisons by dietary therapy status (Table S4). In a between-disease comparison of restricted parents managing dietary therapy, those in the OA group rated “Item 43. Daily home practice of treatments” as more useful (median = 4) than did those in the NICCD group (median = 3) (Table 6).

## 4. Discussion

### 4.1. Health-Related Coping Behaviors of Parents Managing Dietary Therapy During the Transition to Preschool and Group-Based Care

One important finding of this study is that, although parents in the dietary-therapy group reported higher implementation of coping behaviors pertaining to Factor III (Applied Medical Understanding and Communication), they were not consistently rated as highly useful for maintaining health. This discrepancy highlights a potential divergence between the extent to which coping behaviors are implemented and their perceived utility for parental well-being.

Parents of children aged 1–3 years who were not yet enrolled in preschool and required dietary therapy rated “Daily home practice of treatments” as highly useful for maintaining their own health. This finding suggests that, during early childhood, the execution of prescribed treatment within the home may function not only as a disease management task but also as a health-supporting coping behavior for parents.

However, in certain age groups and for certain items, perceived usefulness was lower in the dietary-therapy group than in the non-dietary-therapy group. This pattern was particularly pronounced among parents of 4–6-year-old preschool-enrolled children receiving dietary therapy. Previous studies have identified the transition into preschool or school as a pivotal juncture in disease management and family adaptation [8]. In addition, although many parents adjust to complex disease management and begin to perceive it as the “new normal,” their concerns regarding their child’s social lives—particularly potential exclusion due to dietary restrictions—persist [33]. Our results further suggest that this transition period may also represent a phase in which parents experience heightened difficulty maintaining their own health while adapting to novel caregiving demands and social expectations.

In the subgroup of parents of 4–6-year-old preschool-enrolled children, lower usefulness ratings were observed not only for Factor III items but also for several coping behaviors pertaining to Factor I (e.g., “Investing myself in my children,” “Telling myself that I have many things I should be thankful for”) and Factor II (e.g., “Keeping myself in shape and well groomed,” “Building close relationships with people,” and “Developing myself as a person”). Rather than indicating a diminished importance of family cohesion or social resources, these findings may reflect challenges in recognizing or accessing coping behaviors that effectively support parental health at this developmental stage. Alternatively, parents may rely on coping strategies that are not adequately captured by the scale used in this study.

Children’s autonomy and self-assertion increase in the preschool period, necessitating parents to respond to more complex developmental needs. In addition, the absence of a home-care subgroup among 4–6-year-olds in this study suggests that many children in this age range are expected to participate in group-based settings, reflecting a sociocultural context in which such participation is the norm. Combined with the intense demands associated with group-based care settings—such as coordination with educational institutions and adaptation to shared food environments—these developmental and environmental factors may attenuate the extent to which home-based treatment practices directly contribute to parental health maintenance.

Together, these findings indicate that support for parents managing dietary therapy should not be limited to facilitating treatment implementation alone but should also consider strategies for maintaining their own physical and psychological health amid developmental and environmental transitions. These interpretations are exploratory and hypothesis-generating. To date, little is known about how parents adapt their health-related coping behaviors to maintain their own health across different stages of early childhood development. The mechanisms by which parents modify, prioritize, or struggle to sustain coping behaviors as children move through developmental transitions have not been well characterized. Future studies incorporating qualitative approaches or additional outcome measures are warranted to more comprehensively capture parents’ health-related coping strategies and lived experiences and to identify the forms of support that most effectively promote parental health in group-based care settings.

### 4.2. Health-Related Coping Behaviors of Parents Managing Dietary Therapy Across Disease Categories

This study showed that parents of children with OA who require dietary therapy perceived “Daily home practice of treatments” as a useful health coping behavior. Thus, routine disease management tailored to the child’s medical needs may serve as a factor that supports parents’ health perceptions and behaviors. This finding suggests that in acute metabolic conditions, such as OA, disease management adapted to medical urgency may function as a core health-related coping behavior for parents, rather than being a peripheral daily activity. This interpretation is consistent with previous research suggesting that parental coping strategies, such as positive thinking, may contribute to improvements in parental QOL [34]. Despite increasing parental burden, disease management may—when functioning effectively—support parental health.

Particularly in acute-type disorders, unlike non-acute AA conditions where prevention of chronic onset is paramount, parents face the urgent medical necessity of avoiding acute exacerbations. Thus, treatment adherence is more likely to become the core of parental health-related coping behaviors. Preventing acute episodes is directly linked to stability of family life and is a critical determinant in safeguarding the affected child’s well-being and parents’ physical and mental health. Accounting for the cultural context in Japan, where the long-standing syncretization of Shinto and Buddhism has diminished the explicit recognition of religious belief [35], given this sense of urgency, trust in medical care, such as “Believing the hospital acts in our interest,” may contribute more to maintaining parents’ psychological health than spiritual coping strategies, such as “Believing in God.”

The implementation rate of health coping behaviors within Factor III was particularly high among parents in the dietary-therapy group, suggesting that their engagement is underpinned by trust in healthcare providers and expectations for supportive relationships. Patient education in Japan is largely delivered through individualized instruction provided by attending physicians and dietitians, and reimbursement regulations specify time requirements to ensure the quality of dietary counseling. However, the perceived usefulness of medical information is not always rated highly, and reading informational materials does not necessarily contribute sufficiently to parental health. In Japan, patient-oriented pamphlets and books are available for conditions such as PKU and NICCD. However, for rarer disorders, such as OA, resources are less developed, creating disparities in the availability of accessible information across disorders. Therefore, practical dietary-therapy information that directly supports implementation in the home should be enhanced; for example, menu suggestions informed by the experiential knowledge of families facing similar challenges. These observations are consistent with the notion that coping behaviors in this domain are strongly embedded in healthcare interactions and may be shaped by the quality and accessibility of disease-specific educational resources. Importantly, such coping behaviors are not only behavioral practices but may also be linked to parental perceptions of overall well-being.

However, evidence from previous studies on coping strategies has been inconsistent across metabolic disorders. Studies using the Brief-Coping Orientation to Problem Experienced inventory in IEM requiring dietary restriction (excluding PKU) have reported associations with QOL [7], whereas studies using the CHIP in PKU have found no significant relationship with QOL [36]. These differences may not only reflect the measurement tools used but also differences in disease characteristics between PKU and other metabolic conditions. In conditions such as PKU, where long-term management is relatively stable, coping behaviors may become routinized and embedded in everyday life, making their differences less likely to be reflected in QOL. In contrast, acute metabolic disorders characterized by episodic exacerbations involve greater variability and uncertainty in disease management, with daily care relying more heavily on parental judgment and actions. Consequently, under such conditions, parental health coping behaviors may have a more direct impact on QOL.

These interpretations should be considered within broader social and institutional contexts, in which parental health-related coping behaviors are embedded. In Japan, universal health coverage and social welfare systems provide a baseline level of security for daily living and access to medical care, even in situations of limited income or educational attainment. Consequently, these systems may help ensure a minimum foundation for maintaining parental health, which may partially attenuate the influence of socioeconomic factors that were not assessed in this study.

### 4.3. Study Limitations and Future Directions

This study has some methodological limitations. It was designed as an exploratory investigation targeting rare diseases and was not intended to establish causal relationships or to generate precise estimates of effect sizes. First, although the survey was conducted among all eligible families registered with the JaSMIn, the number of participants was restricted to certain diagnostic and age-based subgroups, which constrained statistical power.

Second, this study reconstructed and utilized a subset of items from an existing scale, and different results might have been obtained had the original scale been employed. Therefore, there are limitations in comparing our findings with those of other studies when characterizing the coping profiles of parents raising children diagnosed with IEM.

Third, because multiple comparisons were performed across questionnaire items and subgroups, the potential for type I error cannot be definitively ruled out. Accordingly, the findings should be interpreted as hypothesis-generating rather than confirmatory.

Future research should extend these findings using longitudinal designs to examine how parental health-related coping behaviors evolve as children progress through different developmental stages. Qualitative approaches may also help capture coping strategies and lived experiences that are not fully represented by standardized questionnaires. In addition, studies incorporating broader contextual and clinical variables, such as caregiving demands, social support systems, and work–life balance, will be important for identifying support strategies that promote parental health, particularly in group-based care settings. Future studies using larger samples, longitudinal designs, and more comprehensive adjustment for contextual and clinical factors will be needed to further elucidate determinants of parents’ health-related coping behaviors.

## 5. Conclusions

This study involved an exploratory investigation of the health-related coping behaviors of parents raising children diagnosed with IEM. Through exploratory subgroup analyses, we found that, among parents of 1–3-year-old children who were not yet enrolled in preschool, “Daily home practice of prescribed treatments for the child” was perceived as a useful health coping behavior for parents. Therefore, reliable and consistent treatment carried out in the home may serve as a foundation that supports not only the child’s health but also the health of the parent. Parents of children diagnosed with acute-type disorders appeared more likely to prioritize medically oriented coping strategies, and may encounter challenges in engaging in social activities, such as building relationships outside the family.

Collectively, these findings provide preliminary insight into how parents’ health-related coping behaviors may vary according to the child’s developmental stage and disease characteristics. These findings may inform future support strategies and intervention research aimed at improving disease management and parental well-being.

## Figures and Tables

**Table 1:** Coping Health Inventory for Parents items included after cognitive debriefing (21 items).

Subscale	Items (Included/Excluded)
**I. Maintaining Family Integration, Cooperation, and an Optimistic Definition of the Situation**	**Included items**
	2. Investing myself in my child(ren)
	5. Telling myself that I have many things I should be thankful for
	6. Building a closer relationship with my spouse
	7. Talking over personal feelings and concerns with spouse
	9. Believing in God
	11. Believing that my child is getting the best medical care possible
	13. Doing things together as a family (involving all members of the family)
	14. Trusting my spouse (or former spouse) to help support me and my child(ren)
	18. Believing that the medical center/hospital has my family’s best interest in mind
	**Excluded items**
	1. Believing that my child(ren) will get better
	3. Doing things with my child(ren)
	4. Believing that things will always work out
	8. Doing things with family relatives
	10. Taking good care of all the medical equipment at home
	12. Trying to maintain family stability
	15. Showing that I am strong
	16. Getting other members of the family to help with chores and tasks at home
	17. Having my child with the medical condition seen at the clinic/hospital on a regular basis
	19. Encouraging child(ren) with medical conditions to be more independent
**II. Maintaining Social Support, Self-Esteem, and Psychological Stability**	**Included items**
	25. Allowing myself to get angry
	27. Concentrating on hobbies (art, music, jogging, etc.)
	28. Working, outside employment
	30. Keeping myself in shape and well groomed
	36. Building close relationships with people
	37. Develop myself as a person
	**Excluded items**
	20. Involvement in social activities (parties, etc.) with friends
	21. Being able to get away from the home care tasks and responsibilities
	22. Getting away by myself
	23. Eating
	24. Sleeping
	26. Purchasing gifts for myself and/or other family members
	29. Becoming more self-reliant and independent
	31. Talking to someone (not professional counselor/doctor) about how I feel
	32. Engaging in relationships and friendships which help me to feel important and appreciated
	33. Entertaining friends in our home
	34. Investing time and energy in my job
	35. Going out with my spouse on a regular basis
**III. Understanding the Health Care Situation Through Communication with Other Parents and Consultation with the Health Care Team**	**Included items**
	38. Talking with other parents in the same type of situation and learning about their experiences
	39. Talking with the medical staff (nurses, social workers, etc.) when we visit the medical center
	41. Reading more about the medical problem which concerns me
	42. Explaining our family situation to friends and neighbors so they will understand us
	43. Being sure prescribed medical treatments for child(ren) are carried out at home on a daily basis
	45. Talking with the doctor about my concerns about my child(ren) with the medical condition
	**Excluded items**
	40. Reading about how other persons in my situation handled things
	44. Talking with other individuals/parents in my same situation

Complement to item 2: Investing myself in my child(ren) (time, money, effort, etc.). Complement to item 28: Working, outside employment (including volunteers).

**Table 2:** Respondent characteristics.

		*n*	%
**Relationship with the child**			
	Mother	176	87.6
	Father	20	10.0
	Parents	5	2.5
**Child’s age (y),** (mean, SD)		**6.52**	**3.2**
0	Home care	6	3.0
1–3	Home care	22	10.9
	Childcare and kindergarten	3	1.5
	Nursery school	16	8.0
	Kindergarten	3	1.5
4–6	Home care	2	1.0
	Childcare and kindergarten	9	4.5
	Nursery school	24	11.9
	Kindergarten	23	11.4
	Regular class	6	3.0
	Special needs school	1	0.5
7–12	Home care	3	1.5
	Regular class	63	31.3
	Special needs class	7	3.5
	Special needs school	10	5.0
	No response	3	1.5
**Number of siblings** (mean, SD)		**1.12**	**1.0**
**Birth order**			
	1st	105	52.2
	2nd	74	36.8
	3rd	19	9.5
	4th	3	1.5
**Diagnostic category**			
**Amino acid metabolism disorder (non-acute type)**		**40**	**19.9**
	Phenylketonuria	30	14.9
	Hyperphenylalaninemia	3	1.5
	Homocystinuria	3	1.5
	Hypermethioninemia	3	1.5
	Tyrosinemia type I	1	0.5
**Amino acid metabolism disorder (acute type)**		**7**	**3.5**
	Maple syrup urine disease	5	2.5
	Nonketotic hyperglycinemia	2	1.0
**Urea cycle disorder (non-acute type)**		**24**	**11.9**
	Citrullinemia (neonatal intrahepatic cholestasis caused by citrin deficiency)	24	11.9
**Urea cycle disorder (acute type)**		**27**	**13.4**
	Ornithine transcarbamylase deficiency	10	5.0
	Citrullinemia type I	8	4.0
	Carbamyl phosphate synthase I deficiency	6	3.0
	Argininosuccinic aciduria	3	1.5
**Organic acid metabolism disorders**		**58**	**28.9**
	Methylmalonic acidemia	15	7.5
	Propionic acidemia	19	9.5
	Isovaleric acidemia	3	1.5
	Glutaric acidemia type 1	7	3.5
	3-methylcrotonylglycinuria	10	5.0
	Multiple carboxylase deficiency	3	1.5
	β-ketoacyl-CoA lyase deficiency	1	0.5
**Fatty acid metabolism disorder**		**31**	**15.4**
	Medium-chain acyl-CoA dehydrogenase deficiency	10	5.0
	Long-chain acyl-CoA dehydrogenase deficiency	14	7.0
	Carnitine palmitoyltransferase I deficiency	2	1.0
	Carnitine palmitoyltransferase II deficiency	1	0.5
	Glutaric aciduria type II	1	0.5
	Carnitine transporter disorder	3	1.5
**Carbohydrate metabolism disorder**		**10**	**5.0**
	Galactosemia	10	5.0
**Other disorders**		**4**	**2.0**
	Inborn error of lipid metabolism	1	0.5
	Cystinuria	1	0.5
	Undiagnosed	2	1.0

**Table 3:** Implementation status of parental health-related coping behavior: dietary therapy vs. non-dietary therapy in the not-enrolled group aged 1–3 years.

	Non-Dietary Therapy (*n* = 7)			Dietary Therapy (*n* = 15)			
	A	B	C	A	B	C	
Domain I							
Item 2	3 (42.9)	1 (14.3)	3 (42.9)	0	1 (6.7)	14 (93.3)	**
Item 5	6 (85.7)	1 (14.3)	0	5 (33.3)	0	10 (66.7)	**
Item 11	2 (28.6)	1 (14.3)	4 (57.1)	0	1 (6.7)	14 (93.3)	*
Item 18	3 (42.9)	0	4 (57.1)	0	0	15 (100.0)	**
Domain III							
Item 38	4 (57.1)	2 (28.6)	1 (14.3)	3 (20.0)	2 (13.3)	10 (66.7)	*
Item 42	6 (85.7)	0	1 (14.3)	3 (20.0)	2 (13.3)	10 (66.7)	**
Item 43	5 (71.4)	0	2 (28.6)	1 (6.7)	0	14 (93.3)	**

Note. Only the results for the items showing significant differences between groups are presented. Full results for all items are provided in Table S1. Implementation status: A, “Choose not to”; B, “Not possible”; C, “Used” (values in parentheses indicate the percentage of respondents). Positive association based on the Cochran–Mantel–Haenszel Test (analysis of variance statistic): * *p* < 0.05, ** *p* < 0.01.

**Table 4:** Usefulness ratings of parental health-related coping behaviors: dietary vs. non-dietary therapy in the preschool-enrolled group aged 4–6 years.

	Non-Dietary Therapy		Dietary Therapy		
	*n*	Median (IQR)	*n*	Median (IQR)	
Domain I					
Item 2	19	4.0 (3–4)	24	3.0 (2–4)	*
Item 5	16	4.0 (3–4)	15	2.0 (2–3)	**
Domain II					
Item 30	19	3.0 (3–4)	24	3.0 (2–3)	*
Item 36	22	3.5 (3–4)	22	2.5 (2–3)	**
Item 37	24	4.0 (3–4)	26	3.0 (2–4)	*

Note. Only the results for the items showing significant differences between groups are presented. Full results for all items are provided in Table S2. *p*-values were calculated using the Wilcoxon rank-sum test to compare the dietary-therapy and non-dietary-therapy groups within each age/enrolled group: * *p* < 0.05, ** *p* < 0.01; IQR, interquartile range.

**Table 5:** Implementation status of parental health coping behavior: dietary therapy vs. non-dietary therapy in the organic acid metabolism group.

	Non-Dietary Therapy (*n* = 28)			Dietary Therapy (*n* = 30)			
	A	B	C	A	B	C	
Domain I							
Item 9	19 (67.9)	0	9 (32.1)	27 (90.0)	2 (6.7)	1 (3.3)	†
Item 18	5 (17.9)	2 (7.1)	21 (75.0)	1 (3.3)	1 (3.3)	28 (93.3)	*
Domain III							
Item 38	19 (67.9)	3 (10.7)	6 (21.4)	7 (23.3)	6 (20.0)	17 (56.7)	**
Item 39	17 (60.7)	3 (10.7)	8 (28.6)	9 (30.0)	1 (3.3)	20 (66.7)	**
Item 41	16 (57.1)	3 (10.7)	9 (32.1)	6 (20.0)	9 (30.0)	15 (50.0)	*
Item 42	17 (60.7)	1 (3.6)	10 (35.7)	7 (23.3)	2 (6.7)	21 (70.0)	**
Item 43	10 (35.7)	0	18 (64.3)	1 (3.3)	1 (3.3)	28 (93.3)	**

Note. Only the results for the items showing significant differences between groups are presented. Full results for all items are provided in Table S3. Implementation status: A, “Choose not to”; B, “Not possible”; C, “Used” (values in parentheses indicate the percentage of respondents). Positive association based on the Cochran–Mantel–Haenszel Test (analysis of variance [ANOVA] statistic): * *p* < 0.05, ** *p* < 0.01. Negative association based on the Cochran–Mantel–Haenszel Test (ANOVA statistic): † *p* < 0.05.

**Table 6:** Disease group-based comparison of parental health-related coping behavior usefulness ratings in dietary therapy vs. non-dietary therapy.

	NICCD					OA					Steel-Dwass Test
	Non-Dietary Therapy		Dietary Therapy		WRST	Non-Dietary Therapy		Dietary Therapy		WRST	
	*n*	Median	*n*	Median		*n*	Median	*n*	Median		
Domain II											
Item 27	3	4.0 (4–4)	18	3.0 (2–4)	*	21	4.0 (3–4)	22	3.0 (2–4)		
Item 36	4	4.0 (2.5–4)	16	3.0 (2–3)		22	4.0 (3–4)	18	3.0 (2–4)	*	
Domain III											
Item 41	2	2.5 (2–3)	15	3.0 (2–3)		9	3.0 (3–3)	15	3.0 (2–3)	*	
Item 42	3	3.0 (3–4)	11	3.0 (3–4)		10	3.5 (3–4)	21	3.0 (2–3)	*	
Item 43	3	3.0 (3–4)	17	3.0 (3–4)		18	4.0 (3–4)	28	4.0 (3–4)		**

Note. Only the results for the items showing significant differences between groups are presented. Full results for all items are provided in Table S4. *p*-values were calculated using the Wilcoxon rank-sum test (WRST) to compare the dietary-therapy and non-dietary-therapy groups within each disease group: * *p* < 0.05. Steel–Dwass multiple comparison test among five groups (comparison among disease groups within the diet group) showed a significant difference between the NICCD and OA groups: ** *p* < 0.05.

## Data Availability

The data presented in this study are available on request from the corresponding author due to privacy or ethical restrictions.

## Supplementary Materials

The following supporting information can be downloaded at https://www.mdpi.com/article/10.3390/ijns12020032/s1. Table S1: Age/enrollment group-based comparison of parental health coping behavior implementation status in dietary therapy vs. non-dietary therapy; Table S2: Age/enrollment group-based comparison of parental health coping behavior usefulness ratings in dietary therapy vs. non-dietary therapy; Table S3: Disease group-based comparison of parental health coping behavior implementation status in dietary therapy vs. non-dietary therapy; Table S4: Disease group-based comparison of parental health coping behavior usefulness ratings in dietary therapy vs. non-dietary therapy.

## Abbreviations

The following abbreviations are used in this manuscript:

AA	Amino acid metabolism disorders
CHIP	Coping Health Inventory for Parents
FA	Fatty acid metabolism disorders
IEM	Inborn errors of metabolism
JaSMIn	Japan Registration System for Metabolic and Inherited Diseases
NICCD	Neonatal intrahepatic cholestasis caused by citrin deficiency
OA	Organic acid metabolism disorders
PA	Propionic acidemia
PKU	Phenylketonuria
QOL	Quality of life
QR	Quick response
